# Neonatal incontinentia pigmenti: Six cases and a literature review

**DOI:** 10.3892/etm.2014.2011

**Published:** 2014-10-09

**Authors:** YANG YANG, YAN GUO, YING PING, XIAO-GUANG ZHOU, YONG LI

**Affiliations:** Department of Neonates, Nanjing Children’s Hospital of Nanjing Medical University, Nanjing, Jiangsu 210008, P.R. China

**Keywords:** incontinentia pigmenti, neonate, autoimmune disease, multiple malformations, Chinese

## Abstract

The aim of this study was to retrospectively analyze the cases of six infants with incontinentia pigmenti (IP) in the Department of Neonates and compare their data with 60 cases of IP reported in the available Chinese literature, in order to determine the clinical characteristics and outcomes of neonatal IP in China. The majority of the cases were located near the eastern and southern coasts of China, and ~98.5% of IP cases occurred within 1 week of birth. The majority of the babies with IP were term infants. Twelve cases had a positive family history of IP. The mothers of 10 patients had a history of recurrent spontaneous abortions, and the mothers of five patients had infectious or autoimmune diseases during pregnancy. Cutaneous manifestations were shown at stage I in 59 cases, at stage II in 28 cases and at stage III in three cases (multiple stages were recorded in certain cases). Neurological changes occurred in 18 cases and ocular changes were observed in 12 cases. The toxoplasmosis, rubella, cytomegalovirus and herpes simplex (TORCH) test showed positive results in three cases; autoantibody positivity was found in three cases and high blood eosinophil levels were observed in 20 cases. Brain scans revealed positive results in 16 cases and complications were observed in 21 cases. Thirty-four cases were followed for 1–6 months, six cases for 7–12 months and 17 cases for 13–84 months. Among these cases, 34 exhibited no evidence of recurrence. Five patients, including one male, succumbed in the long course of the follow-up. Two IP cases persisted after five years of follow-up. The data from the present study may reflect the characteristics of IP in the Chinese population and provide useful information for the diagnosis and treatment of IP by dermatologists and neonatologists.

## Introduction

Incontinentia pigmenti (IP), also known as Bloch-Sulzberger syndrome or nuclear factor-κB essential modulator (NEMO) syndrome, is an uncommon skin disorder characterized by an X-linked dominant inheritance in the majority of cases (1). This syndrome is known as a multi-systemic disease, which seriously affects skin, teeth, hair and the central nervous system. Among these clinical signs, the most notable symptom during the neonatal period is skin lesions, which are characterized by a linear pattern of erythema with vesicles and bullae appearing on the extremities and trunk following birth.

The pathogenesis of IP syndrome has been defined as a primary immunodeficiency with immune and non-immune manifestations (2). Previous clinical studies have reported the involvement of IP syndrome in inflammatory and autoimmune diseases, including atypical enterocolitis, Behçet’s syndrome and rheumatoid arthritis (3–5). The immunodeficiency is particularly severe in surviving males, who are typically affected by rare types of NEMO mutations that differ from the more common deletion. Therefore, this mode of inheritance is associated with male lethality *in utero*, and the majority of live adult patients are female.

Since IP is a rare syndrome, multicenter or large-scale clinical trials are impracticable. Several studies in China have reported IP syndrome in neonates (6,7); however, a number of these studies lacked a systematic summary and detailed information of the different cases. Therefore, in the present study, six cases from the Nanjing Children’s Hospital of Nanjing Medical University (Nanjing, China) were described and the available cases of neonatal IP in China were analyzed. The results from this study may reflect the characteristics of the Chinese population, in addition to providing useful information for pediatricians.

## Materials and methods

### Data collection

A total of six patients diagnosed with IP in the Department of Neonates were reviewed, and the data were compared with those from previous studies in China. The Institutional Review Board at Nanjing Children’s Hospital of Nanjing Medical University approved this study. Parental informed consent was obtained using an Institutional Review Board approved consent form. The diagnosis of IP was confirmed in all cases using the diagnostic criteria presented by Landy and Donnai (1), including clinical manifestations, laboratory data and imaging information. All previous cases were reported in Chinese journals between January 1993 and December 2013. These reports were found through a search using three Chinese medical journal search engines [Cqvip (http://www.cqvip.com/), Wanfang Data (http://www.wanfangdata.com/) and Science China (http://www.scichina.com/)] using the keywords ‘Incontinentia Pigmenti’ and ‘Infant’, and were then retrospectively analyzed.

## Results

### Report of six cases

#### History

Between November 2003 and November 2013, six patients with IP, including one male, were referred to the Nanjing Children’s Hospital of Nanjing Medical University due to different types of skin eruption (Tables I and II). Age on admission ranged between 4 h and 28 days. Gestational age and birth weight varied between 37 and41 weeks, and 2,600 and 4,150 g, respectively. No significant family history of IP was noted in any of the six patients. The mothers of patients 2, 4 and 6 had abnormal amniotic fluid, including oligohydramnios, polyhydramnios or turbidity. In addition, the mothers of patients 2 and 6 suffered from influenza and lupus separately during pregnancy. The mothers of patients 3 and 6 had a positive history of repeated abortion.

#### Clinical manifestations

Three patients showed typical vesicular-stage skin changes on admission, with linear or ink-like vesicular eruption on the extremities from birth. This skin lesion often contained translucent or transparent material inside, and was surrounded by redness and swelling on the surface of skin. A number of the eruptions merged together or ulcerated with exudation. The main manifestations in the other three infants were verrucous lesions on admission. A few blisters were observed, as well as verrucous hyperplasia with thickening of the texture and surface. Subsequent skin biopsies all showed typical histological changes, including aggregation of eosinophils (EOSs). In addition, the rash imprint and smear exhibited infiltration of numerous EOSs (10.3–22.0%; data not shown).

In addition to skin lesions, neurological changes, ocular abnormalities and alopecia were the three other key symptoms that were observed during the physical examination. Patient 4 suffered from several transient seizures following admission (at 4 days of age). The seizures were of the grand mal type, with mouth twitching, staring eyes and stiffness of extremities. Decreased muscle tone was found in patients 2 and 4. Alopecia was also observed in patient 2, who showed sparse, thinning hair and eyebrows following birth. Patient 5 was diagnosed with retinopathy following a detailed fundus examination. All six patients had varying degrees of complications. Patients 1 and 6 had myocardial damage and congenital heart disease, while patients 2, 3, 4 and 5 suffered from infections of varying severity. Notably, patient 2 additionally suffered from an autoimmune disease, lupus.

#### Auxiliary examination

A series of auxiliary examinations associated with IP were performed following admission. Routine chest and abdominal X-rays showed normal results in these six infants. Although cerebrospinal fluid tests were negative, brain ultrasounds revealed intracranial hemorrhages (ICHs) in all six patients. Notably, as well as ICH (stage III), high signal intensity lesions of the brain stem and frontal and occipital lobes were observed in patient 4 using magnetic resonance imaging, indicating cerebral edema and subcortical hemorrhage. The electroencephalogram revealed rhythmic spikes and slow waves in this patient.

Compared with patients 5 and 6, patient 1 showed lower immunoglobulin G levels and a higher natural killer cell ratio, as indicated by humoral immune function and cellular immune function tests. White blood cell (WBC) and EOS counts were recorded on admission and at discharge. WBC counts on admission ranged between 9.25×10^9^/l and 21.13×10^9^/l, and between 9.59×10^9^/l and 17.91×10^9^/l at discharge. EOS counts on admission ranged between 0.70×10^9^/l (5.1%) and 5.33×10^9^/l (25.2%), and between 0.50×10^9^/l (5.2%) and 4.61×10^9^/l (33.0%) at discharge. In patients 5 and 6, C-reactive protein levels were normal on admission while moderately elevated at discharge. In addition, autoantibody tests showed positive results in patients 1, 4 and 6, and cytomegalovirus (CMV) infection was found in patients 1 and 3 using the toxoplasmosis, rubella, CMV and herpes simplex (TORCH) test.

#### Therapeutic procedure

Cod-liver oil was used for all patients to alleviate the skin symptoms. Mupirocin ointment was also applied to prevent cutaneous infection until discharge. In patients 1 and 2, a sharp decrease in the EOS ratio was observed following symptomatic treatments. For patients 3 and 4, antibiotics were administered to treat bacterial infection according to the positive blood culture results. In view of the myocardial damage and liver function damage of patients 1, 5 and 6, creatine phosphate sodium and glutathione were administered for organ protection. For patient 4, phenobarbital and mannitol were applied together to control the seizures and alleviate encephaledema.

#### Outcome and prognosis

The symptoms of the six patients were improved to varying degrees at discharge. Patient follow-up ranged between 2 and 14 months with an average of 6.58 months. With the exception of patient 5, the patients remained alive at the end of follow-up. However, patients 4 and 6 presented with recurrence following discharge.

### Literature review

#### Distribution of neonatal IP in China

**Figure 1:**
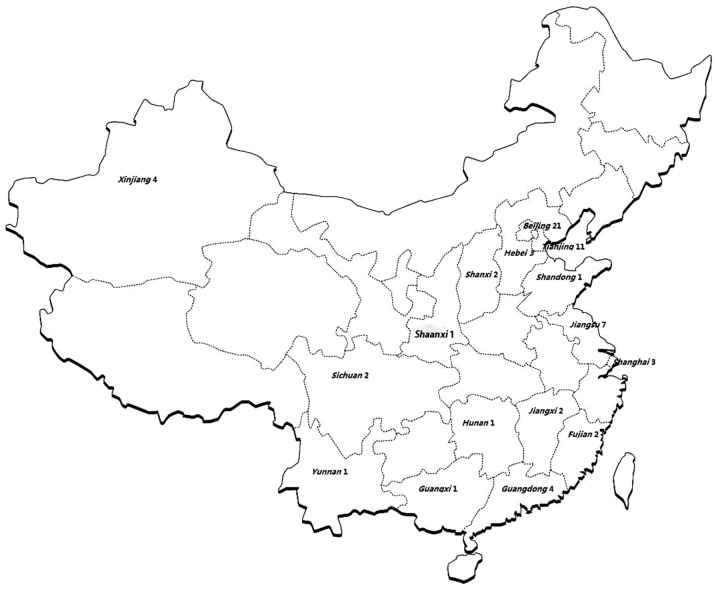
Distribution of neonatal incontinentia pigmenti in China.

Among the 163 cases of IP reported previously in Chinese journals, a total of 66 cases occurring during the neonatal period were available for analysis (Tables III–V) (6–36). Among them, 21 cases were from Beijing, 11 cases were from Tianjin, seven cases were from Jiangsu province, four cases were from Guangdong and Xinjiang provinces, respectively, three cases were from Shanghai and Hebei province, respectively, two cases were from Shanxi, Jiangxi, Fujian and Sichuan provinces, respectively, and one case was from Yunnan, Guangxi, Hunan, Shaanxi and Shandong provinces, respectively. The majority of the provinces and cities were located near the eastern and southern coasts of China. Fig. 1 shows the distribution of the 66 cases.

#### Clinical manifestations of the 66 cases in China

- Birth weight and gender. The mean gestational age at delivery was 38.09±1.38 weeks (median, 38.09 weeks; range, 34–41 weeks). The average weight was 3,156.45±460.73 g (median, 3,156.45 g; range, 2,500–4,350 g). Among the 66 infants, three patients (4.9%, 3/61) were <37 weeks. The patients included nine males (13.6%) and 57 females (86.4%), and the female-to-male ratio was 6.33:1.
- Family history. A total of 12 cases (21.8%, 12/55) had a positive family history of IP. The mothers of 10 infants with IP (28.6%, 10/35) had a history of recurrent spontaneous abortions. The mothers of five patients (10.9%, 5/46) had infectious or autoimmune diseases during pregnancy, and abnormal amniotic fluid was observed in six cases (21.4%, 6/28).
- Onset of symptoms. Clinical manifestations were observed <1 week from birth in 65 cases (98.5%), and >1 week in one case (1.5%). Cutaneous manifestations were observed at stage I in 59 cases (65.6%), at stage II in 28 cases (31.1%) and at stage III in three cases (3.3%) (multiple stages were recorded in certain cases). No cutaneous manifestations were observed at stage IV.
- Major manifestations of IP. In addition to the typical skin lesions, neurological changes occurred in 18 cases (27.3%), ocular changes were observed in 12 cases (30.0%, 12/40) and alopecia was observed in five cases (8.9%, 5/56).

### Auxiliary examination results of the 66 patients with IP

The results from the TORCH test were positive in three cases (10.3%, 3/29). Tests for autoantibodies were positive in three cases (30.0%, 3/10) and high blood EOS levels were observed in 20 cases (95.2%, 20/21). Brain scans revealed positive results in 16 cases (39.0%, 16/41), including edema and hemorrhages.

### Complications, outcomes and follow-up

During the neonatal period, complications were observed in 21 cases (32.3%, 21/65). A total of 34 cases were followed for 1–6 months, six cases for 7–12 months and 17 cases for 13–84 months. Among them, 34 cases (59.6%) had no evidence of recurrence (data not shown). Nine patients were not followed up subsequent to discharge due to social and family factors. Five patients, including one male, succumbed during the follow-up. IP was found to persist in two cases after five years.

## Discussion

IP is a rare hereditary disease, and the clinical and epidemiological characteristics of IP in China remain almost unknown compared with the fairly abundant historical data from other countries. The incidence of IP is ~1/500,000 individuals per year worldwide, and 50–96% of cases have a positive family history (37). However, the specific incidence of this disease is still not clear in China, even as a rough estimate. Therefore, in the present study, the available data on Chinese patients with IP were collected and analyzed.

In the present review, 60 cases were analyzed. The majority of the IP cases were located near the eastern and southern coasts of China, including Beijing, Tianjin and Jiangsu, Guangdong and Hebei provinces; however, a small number of cases were also reported in other regions. To the best of our knowledge, this is the first report involving the distribution of IP in China. The reason for this distribution disequilibrium is currently unknown; however, economic and social factors, as well as the awareness and misdiagnosis of this disease in different areas of China, may play a role in the pathogenesis of this unusual disease in China.

Among the cases analyzed, females accounted for 86.4% (57/66), while males constituted 13.6% (9/66). Consistent with an international study (41), the majority of the patients were female. However, data from a recent large-sample study in Serbia revealed a male-to-female ratio of 91.83 to 5.85% (~15.70:1) (38), which is markedly higher than the ratio found in the present study (6.33:1). Furthermore, in the present study, the majority of the patients with IP were term babies (58/61) and had a normal birth weight (51/51), suggesting that IP syndrome is more common in term infants. The reason for this is not clear; however, the mothers of IP patients appear to have a higher chance of miscarriage. Therefore, the mortality of fetuses caused by fetal death and stillbirth is higher in these mothers, which contributes to a lower live birth rate in preterm patients with IP. In the present study, the onset ages were almost all <1 week (65/66), with the majority within 3 days (62/65), which provides a good opportunity for early diagnosis and treatment. However, international studies with accurate data regarding onset age are lacking.

The physical condition of the mothers and the family history were also investigated in this study. According to the present results, abnormal maternal health was observed in five cases (5/46). Notably, patient 2 and the mothers of patients 2 and 5 suffered from lupus erythematosus. In addition, patients 2, 5 and 6 all showed positive results for autoantibodies. As reported by Piccoli *et al* (39), chronic NEMO inhibition has an important role in the development of complex immunological diseases, of which systemic lupus erythematosus (SLE) is often considered the prototype. Therefore, there may be a possible association between IP and certain autoimmune diseases, including SLE. Similar to a previous study (41), a history of abortion was not uncommon in the mothers of the patients with IP (10/35). However, as a result of the family planning policy, numerous Chinese families volunteer to have only one child; therefore, the birth rate is lower than that in foreign countries and the abortion rate appeared lower than the real situation. Familial history is a common characteristic in this syndrome, and, in the present study, a positive rate of ≤21.8% (12/55) was observed. Cases 3 and 4 were particularly noteworthy. These two female infants suffered from IP while their brothers and mothers all appeared to be healthy. This suggested that their fathers were symptomless carriers of the NEMO gene. A similar situation was observed in the father of case 9, who only presented with a mild cutaneous manifestation. Therefore, to a certain degree, male carriers of the NEMO gene can have a nearly normal quality of life. Although the specific mechanism is not yet clear, the International IP Consortium proposed three mechanisms for survival of these males: Hypomorphic alleles, the 47, XXY karyotype (Klinefelter syndrome) and somatic mosaicism (40).

Among the clinical manifestations of IP, skin change is a major diagnostic criteria. In the present study, the constituent ratio was analyzed and skin lesions of stages I and II were found in a large proportion of the cases. It was difficult to distinctly separate the stages of the patients, since the skin lesions of certain patients exhibited the characteristics of more than one stage. Furthermore, several of the cases showed marked melanin incontinence in the skin, which is a characteristic of stage III and not common during the neonatal period (41). Therefore, in clinical practice it is occasionally too difficult to separate the stages clearly based on skin manifestation, and further discussion may be required. In addition to skin lesions, neurological changes, ocular abnormalities and alopecia were the other three key symptoms observed. Hair anomalies were classified as a minor criterion for IP (42). A total of 8.93% cases of alopecia (5/56) were found in the present study, which was significantly lower than the values in the literature (43,44). This phenomenon could be explained due to hair anomalies typically being more common in childhood. Ocular abnormalities, including hyphema, retinal hemorrhage, retinal detachment, optic neuritis, optic atrophy and retinopathy, were observed in 18.0% of the cases. Although retinal anomalies are important components of the diagnostic criteria of IP, it was found that non-retinal ocular anomalies, such as optic neuritis and atrophy, were also not uncommon in patients with IP. Therefore, when making the diagnosis of atypical IP, it is possible that non-retinal ocular anomalies should also be taken into account. Seizure was an important clinical manifestation of neurological changes and was found in 27.27% of patients (18/66), which was near the value from previous reports (38,45). However, it should be noted that findings in the brain scan were quite inconsistent with the clinical manifestations. For instance, certain patients with apparent image abnormalities did not show neurological changes in clinical practice (such as patients 23 and 31), and the reverse was also common (such as patients 23 and 31). Although this contradiction has lacked a reasonable explanation, it may be associated with the time-point of scanning. Clinical manifestations could be inconsistent with imaging results in the early and recovery stages of certain diseases.

In conclusion, this study presents 66 cases of neonatal IP in China. Among these, the majority of patients came from the eastern and southern coasts of China. Approximately 98.5% of IP cases occurred within <1 week of birth, particularly within three days. The majority of the babies with IP were term infants. The incidence of alopecia (8.93%) and the male-to-female ratio (6.33:1) were lower than the values in the international data. Although male patients exhibited a higher mortality rate, they could almost have a normal quality of life. There appears to be a potential association between IP and certain autoimmune diseases, such as SLE and Sjögren’s syndrome. The skin lesions of certain patients exhibited the features of two stages, and could show marked characteristics of stage III, which is not common during the neonatal period. Findings in the brain scan were also relatively inconsistent with the clinical manifestations. Rare, non-retinal ocular anomalies, such as optic neuritis and atrophy, were also found in patients with IP. This indicated that more considerations should be taken into account when making the diagnosis of atypical IP. Clinicians such as dermatologists, neonatologists and neurologists should thus be acquainted with IP, and we suggest that the management of infants with IP requires a skilled pediatric team with experience in the diagnosis and management of IP in this young age group.

## Figures and Tables

**Table I:** Auxiliary examination of six incontinentia pigmenti cases in the Department of Neonates (Nanjing Children’s Hospital of Nanjing Medical University).

Case no.	Eye	Brain ultrasound (ICH stage)	Brain CT/MRI	EEG	X-ray^a^	Skin biopsy	CSF	HIF, IgM/G/A (g/l)	CIF, T/NK/B (%)	AAbs	TORCH	On admission			At discharge		
	
												WBC (10^9^/l)	EOS (10^9^/l)	CRP (mg/dl)	WBC (10^9^/l)	EOS (10^9^/l)	CRP (mg/dl)
1	N	II	N	N	N	T	N	0.15/1.84/0.048	79.80/15.13/3.99	NR	CMV (+)	19.90	3.36	N	9.59	0.50	N
2	N	II	N	N	N	T	N	NR	NR	(+)	(−)	15.37	2.37	N	13.12	0.81	N
3	N	II	N	N	N	T	N	NR	NR	(−)	CMV (+)	14.01	NR	N	NR	NR	NR
4	N	III	Swelling	Abnormal	N	T	N	NR	NR	(−)	(−)	9.25	0.95	N	13.98	4.61	N
5	Ret	II	N	N	N	T	N	0.17/7.79/0.058	77.70/2.25/14.94	(+)	(−)	13.63	0.70	N	16.12	1.11	11
6	N	I–II	N	N	N	T	N	0.17/5.47/0.064	82.90/6.17/4.33	(+)	(−)	21.13	5.33	N	17.91	3.82	9

a Chest and abdominal X-ray.

ICH, intracranial hemorrhage; CT, computed tomography; MRI, magnetic resonance imaging; EEG, electroencephalogram; CSF, cerebrospinal fluid; HIF, humoral immune function; IgM/G/A, immunoglobulin M/G/A; CIF, cellular immune function; T, T cells; NK, natural killer cells; B, B cells; AAbs, autoantibodies; TORCH, toxoplasmosis, rubella, cytomegalovirus and herpes simplex; WBC, white blood cell; EOS, eosinophil; CRP, C-reactive protein; N, normal; T, typical; NR, not reported; Ret, retinopathy.

**Table II:** Clinical manifestations, outcomes and prognosis of six incontinentia pigmenti cases in the Department of Neonates (Nanjing Children’s Hospital of Nanjing Medical University).

Case no.	Age^a^ (days)	Gender	BW (g)	GA (weeks^+days^)	Onset	Amniotic fluid	Maternal health	Abortion history	Familial history	Alopecia	Skin lesion (stage)	Seizure	Muscular tension	Complication	Follow-up (months)	Prognosis
1	28	M	2700	37	*In utero*	Normal	Normal	-	-	(−)	II	(−)	Normal	MD; CHD	5.0	Alive
2	4	F	2600	39^+6^	*In utero*	Oligohydramnios; turbidity	Influenza; lupus			(+)	I	(−)	Reduced	Lupus; CI	6.0	Alive
3	21	F	3400	39	*In utero*	NR	Normal	Twice	Healthy (3 males)	(−)	II	(−)	Normal	Sepsis	3.5	Alive
4	4	F	4150	40	*In utero*	Polyhydramnios; turbidity	Normal	-	Healthy (1 male)	(−)	I	(+)	Reduced	CHD; sepsis	2.0	Alive; recurrences
5	17	F	2900	41	*In utero*	Normal	Normal	-	-	(−)	II	(−)	Normal	InI; LFD	14.0	Dead
6	10	F	3600	38^+4^	*In utero*	Oligohydramnios; turbidity	Lupus	Thrice	-	(−)	I	(−)	Normal	MD; CHD	9.0	Alive; recurrences

a Age on admission.

M, male; F, female; BW, birth weight; GA, gestational age; NR, not reported; MD, myocardial damage; CHD, congenital heart disease; CI, cytomegalovirus infection; InI, intrauterine infection; LFD, liver function damage.

**Table III:** General information on 66 incontinentia pigmenti cases in China.

First author (reference)	Location	Case no. (year)	Gender	BW (g)	GA (weeks)	Onset (day)	Amniotic fluid	Maternal health	Abortion history	Familial history
Cao (6)	Xinjiang	7 (1999)	F	NR	39	1	NR	N	NR	+ (Mother)
		8 (1999)	F	NR	38	2	N	N	NR	−
Liu (7)	Tianjin	9 (2000)	F	2700	37	1	N	N	−	+ (Father)
		10 (2000)	F	3100	38	3	N	N	NR	−
		11 (2000)	F	3350	38	1	N	N	NR	−
		12 (2000)	F	2900	37	1	N	N	−	−
		13 (2000)	F	3500	39	1	N	N	NR	−
		14 (2000)	M	3000	38	1	N	N	NR	−
Fan (8)	Shandong	15 (2001)	F	3000	39	1	N	N	−	−
Liu (9)	Beijing	16 (2002)	F	NR	NR	1	N	N	NR	NR
Chen (10)	Beijing	17 (2004)	M	NR	37	1	NR	N	−	−
Xu (11)	Jiangxi	18 (2004)	F	NR	37	1	NR	NR	−	NR
		19 (2004)	F	NR	37	1	NR	NR	NR	NR
Han (12)	Beijing	20 (2005)	M	NR	38	1	N	Diabetes	+	−
Song (13)	Beijing	21 (2005)	F	NR	NR	3	NR	N	NR	−
Tang (14)	Beijing	22 (2005)	F	3300	39	1	NR	NR	NR	NR
		23 (2005)	F	2800	38	1	NR	NR	NR	NR
		24 (2005)	F	2500	NR	1	NR	NR	NR	NR
		25 (2005)	M	3800	39	6	NR	NR	NR	NR
		26 (2005)	M	3880	38	1	NR	NR	NR	NR
		27 (2005)	M	4050	39	1	NR	NR	NR	NR
Cui (15)	Yunnan	28 (2006)	F	2800	37	3	NR	N	−	−
Liu (16)	Shanxi	29 (2006)	F	NR	34	1	N	N	−	−
Zhang (17)	Beijing	30 (2007)	F	3310	41	1	N	Influenza	−	−
Dong (18)	Hebei	31 (2008)	F	NR	37	3	NR	N	−	−
He (19)	Guangdong	32 (2008)	M	2850	40	1	Oligohydramnios	N	+	−
		33 (2008)	F	3100	38	1	Meconium stained	Influenza	+	−
Zhou (20)	Xinjiang	34 (2009)	F	NR	37	4	NR	N	−	+ (Sister)
		35 (2009)	F	2500	37	1	NR	N	−	+ (Sister)
Tang (21)	Hunan	36 (2009)	M	2850	36	2	N	N	+	−
Li (22)	Hebei	37 (2009)	F	3000	37	1	NR	N	−	−
Wu (23)	Guangxi	38 (2009)	F	3400	37	3	NR	N	−	−
Zhang (24)	Guangdong	39 (2010)	F	3140	39	1	N	N	+	−
Luo (25)	Fujian	40 (2010)	F	NR	NR	1	NR	NR	NR	NR
Hao (26)	Tianjin	41 (2010)	F	2700	38	1	NR	N	NR	+ (Mother)
		42 (2010)	F	2500	37	1	NR	N	−	−
Hao (26)	Tianjin	43 (2010)	F	2900	39	1	NR	N	NR	+ (Sister)
		44 (2010)	F	4100	40	6	NR	N	NR	−
		45 (2010)	F	3200	41	24	NR	N	NR	−
Chen (27)	Sichuan	46 (2010)	F	2780	39	1	Meconium stained	N	+	−
Wang (28)	Shanghai	47 (2010)	F	4350	38	1	N	N	+	−
		48 (2010)	F	2669	38	1	N	N	−	+ (Mother and grandmother)
Li (29)	Shanghai	49 (2010)	F	NR	37	1	NR	N	−	+ (Mother)
Zhao (30)	Jiangsu	50 (2010)	F	NR	NR	1	NR	NR	NR	NR
Yang (31)	Shanxi	51 (2011)	F	3310	41	2	N	N	−	+ (Sister)
		52 (2011)	F	NR	39	1	N	N	−	+ (Sister)
Huang (32)	Fujian	53 (2012)	F	3330	40	1	NR	N	+	−
Zheng (33)	Beijing	54 (2012)	F	2750	37	1	NR	NR	NR	−
		55 (2012)	F	2500	34	1	NR	NR	NR	−
		56 (2012)	F	3310	41	3	NR	NR	NR	−
		57 (2012)	F	3200	39	1	NR	NR	NR	−
		58 (2012)	F	3000	39	1	NR	NR	NR	−
		59 (2012)	F	3000	37	1	NR	NR	NR	−
		60 (2012)	F	3150	37	1	NR	NR	NR	+ (Mother)
		61 (2012)	F	3300	38	3	NR	NR	NR	−
		62 (2012)	F	3950	39	1	NR	NR	NR	−
		63 (2012)	F	3100	37	1	NR	NR	NR	−
Ma (34)	Hebei	64 (2012	F	3000	38	1	NR	N	−	−
Fang (35)	Guangdong	65 (2013)	F	3300	37	3	N	N	−	+ (Grandmother)
Li (36)	Sichuan	66 (2013)	F	3400	38	1	N	N	−	−

F, female; M, male; BW, birth weight; GA, gestational age; NR, not reported; N, normal.

**Table IV:** Clinical manifestations of 66 incontinentia pigmenti cases in China.

First author (reference)	Location	Case no. (year)	Eye	Alopecia	Skin lesion (stage)	Seizure	Complication	Follow-up (months)	Brain scan	Skin biopsy	AAbs	TORCH	EOS on admission, % (×10^9^/l)
Cao (6)	Xinjiang	7 (1999)	NR	(−)	I	(−)	Sepsis	1	NR	T	NR	NR	NR
		8 (1999)	NR	(−)	I–II	(+)	-	1.5	NR	T	NR	NR	NR
Liu (7)	Tianjin	9 (2000)	Optic neuritis	(−)	I	(+)	Pneumonia: jaundice	1.5	(−)	T	NR	NR	17.0 (1.70)
		10 (2000)	-	(−)	I–II	(−)	Jaundice	1.5	(−)	T	NR	NR	NR
		11 (2000)	-	(−)	I–II	(−)	Pneumonia	1.5	(−)	T	NR	NR	NR
		12 (2000)	-	(−)	I–II	(+)	Pneumonia: jaundice	1.5	(−)	T	NR	NR	NR
		13 (2000)	-	(−)	I–II	(−)	Pneumonia	1.5	(−)	T	NR	NR	NR
		14 (2000)	Optic atrophy	(−)	I	(+)	Pneumonia: jaundice	1: mortality	(−)	T	NR	NR	25.0 (4.75)
Fan (8)	Shandong	15 (2001)	NR	(−)	I	(−)	-	Lost	NR	T	NR	NR	30.0 (5.52)
Liu (9)	Beijing	16 (2002)	-	(−)	I–II	(−)	-	1.5	NR	T	NR	NR	NR
Chen (10)	Beijing	17 (2004)	-	(−)	I	(−)	-	1	NR	T	NR	NR	NR
Xu (11)	Jiangxi	18 (2004)	-	(−)	I	(−)	-	36	NR	T	NR	NR	NR
		19 (2004)	NR	(−)	I	(−)	-	1	NR	T	NR	NR	NR
Han (12)	Beijing	20 (2005)	-	(−)	I	(−)	-	1	(−)	T	NR	NR	NR
Song (13)	Beijing	21 (2005)	Retinopathy	(−)	II	(−)	-	2	NR	T	NR	NR	NR
Tang (14)	Beijing	22 (2005)	Optic atrophy	(−)	I	(−)	-	24	NR	T	NR	(−)	NR
		23 (2005)	-	(−)	I	(−)	-	14	(+)	T	NR	NR	NR
		24 (2005)	-	(−)	I	(+)	-	10	(+)	T	NR	(−)	NR
		25 (2005)	Retinopathy	(−)	I–II	(−)	-	10	NR	T	NR	(−)	NR
		26 (2005)	-	(−)	I–III	(−)	-	8	NR	T	NR	NR	NR
		27 (2005)	-	(−)	I–III	(+)	-	2	(+)	T	NR	NR	NR
Cui (15)	Yunnan	28 (2006)	NR	(−)	I	(−)	-	1	(−)	T	NR	(−)	58.2 (11.91)
Liu (16)	Shanxi	29 (2006)	-	(−)	I–II	(−)	Pneumonia	8	NR	T	NR	(+)	2.6 (0.40)
Zhang (17)	Beijing	30 (2007)	-	(−)	I	(−)	-	14	NR	T	(−)	(−)	NR
Dong (18)	Hebei	31 (2008)	NR	(−)	I–II	(−)	Myocardial damage	Lost	(+)	T	NR	NR	NR (0.60)
He (19)	Guangdong	32 (2008)	-	(+)	I	(+)	-	Lost	(+)	T	(−)	(−)	63.0 (19.59)
		33 (2008)	NR	(−)	I–II	(+)	-	1	(−)	T	(−)	(−)	15.0 (2.34)
Zhou (20)	Xinjiang	34 (2009)	NR	(+)	III	(−)	-	36	NR	T	NR	NR	NR
		35 (2009)	NR	(−)	I	(−)	-	3	NR	T	NR	NR	NR
Tang (21)	Hunan	36 (2009)	-	(−)	I–II	(+)	Pneumonia	19	(+)	T	(−)	(−)	14.0 (3.87)
Li (22)	Hebei	37 (2009)	NR	(−)	I–II	(−)	-	1	NR	T	NR	NR	17.6 (2.61)
Wu (23)	Guangxi	38 (2009)	NR	NR	I	(−)	-	1	(−)	T	NR	(−)	21.0 (3.53)
Zhang (24)	Guangdong	39 (2010)	-	(−)	I	(−)	-	Lost	NR	T	NR	(−)	7.3 (NR)
Luo (25)	Fujian	40 (2010)	Hyphema	NR	I–II	(−)	Pneumonia	1	(−)	T	NR	(−)	NR
Hao (26)	Tianjin	41 (2010)	-	(−)	I	(+)	-	2	(−)	A	NR	(−)	NR
Hao (26)	Tianjin	42 (2010)	-	NR	II	(+)	-	2	(−)	T	NR	(−)	NR
		43 (2010)	-	NR	I	(+)	-	2	(+)	T	NR	(−)	NR
		44 (2010)	-	NR	I	(−)	-	2	(−)	T	NR	(−)	NR
		45 (2010)	-	NR	II	(−)	-	2	(−)	T	NR	(−)	NR
Chen (27	Sichuan	46 (2010)	-	(−)	I	(−)	-	Lost	(−)	T	NR	NR	NR
Wang (28	Shanghai	47 (2010)	-	(−)	I	(+)	-	7	(−)	T	(−)	(−)	NR
		48 (2010)	-	(−)	I	(−)	-	4	NR	T	NR	(−)	21.0 (NR)
Li (29)	Shanghai	49 (2010)	NR	NR	I	(−)	-	1	NR	T	NR	NR	NR
Zhao (30)	Jiangsu	50 (2010)	-	(−)	I	(−)	Pneumonia	1.5	NR	T	NR	(−)	7.8 (1.90)
Yang (31)	Shanxi	51 (2011)	-	(+)	I–II	(−)	NR	60	NR	T	NR	NR	21.0 (3.89)
		52 (2011)	NR	NR	I	(−)	-	1	NR	T	NR	NR	35.0 (4.97)
Huang (32)	Fujian	53 (2012)	NR	NR	I	(−)	-	1.5	NR	T	NR	(−)	NR
Zheng (33)	Beijing	54 (2012)	-	(−)	I	(+)	-	18: mortality	(+)	T	NR	NR	NR
		55 (2012)	-	(−)	I–II	(−)	Pneumonia	23	(−)	T	NR	NR	NR
		56 (2012)	-	(−)	I–II	(+)	Pilonidal sinus	21: mortality	(+)	T	NR	NR	NR
		57 (2012)	Retinal hemorrhage	(−)	I	(+)	-	46	(−)	T	NR	NR	NR
		58 (2012)	Retinal detachment	(−)	I	(−)	-	Lost	(+)	T	NR	NR	NR
		59 (2012)	-	(−)	I–II	(+)	-	29: mortality	(+)	T	NR	NR	NR
		60 (2012)	-	(−)	I	(−)	-	28	(+)	T	NR	NR	NR
		61 (2012)	-	(−)	I–II	(−)	Apnea	Lost	(+)	T	NR	NR	NR
		62 (2012)	-	(−)	I–II	(−)	-	84	(+)	T	NR	NR	NR
		63 (2012)	-	(+)	I–II	(−)	-	42: mortality	(+)	T	NR	NR	NR
Ma (34)	Hebei	64 (2012)	NR	(−)	I	(−)	-	Lost	NR	A	NR	NR	NR
Fang (35)	Guangdong	65 (2013)	-	(−)	I–II	(−)	-	24	(−)	T	NR	(−)	NR
Li (36)	Sichuan	66 (2013)	NR	NR	I–II	(−)	-	Lost	NR	T	NR	(−)	56.9 (10.12)

NR, not reported; T, typical; A, atypical; AAbs, autoantibodies; EOS, eosinophil; TORCH, toxoplasmosis, rubella, cytomegalovirus and herpes simplex.

**Table V:** Summary of the clinical manifestations of the 66 incontinentia pigmenti cases.

Category	n (%)
Gender	
Female	57/66 (86.4)
Male	9/66 (13.6)
Gestational age	
Term	58/61 (95.1)
Pre-term	3/61 (4.9)
History of incontinentia pigmenti	
Familial	12/55 (21.8)
Sporadic	43/55 (78.2)
Onset	
<1 week	65/66 (98.5)
>1 week	1/66 (1.5)
Amniotic fluid	
Normal	22/28 (78.6)
Abnormality	6/28 (21.4)
Abortion history	
Positive	10/35 (28.6)
Negative	25/35 (71.4)
Cutaneous manifestations	
Stage I	59/90 (65.6)
Stage II	28/90 (31.1)
Stage III	3/90 (3.3)
Stage IV	0 (0)
Extracutaneous manifestations	
Neurological	18/66 (27.3)
Ocular	9/50 (18.0)
Alopecia	5/56 (8.9)
Histological features	
Typical	64/66 (97.0)
Atypical	2/66 (3.0)
TORCH	
Positive	3/29 (10.3)
Negative	26/29 (89.7)
Autoantibodies	
Positive	3/10 (30.0)
Negative	7/10 (70.0)
Blood eosinophilia	20/21 (95.2)

TORCH, toxoplasmosis, rubella, cytomegalovirus and herpes simplex.
